# Longitudinal transactional relationships between caregiver and child mental health during the COVID-19 global pandemic

**DOI:** 10.1186/s13034-021-00422-1

**Published:** 2021-11-15

**Authors:** Emily L. Robertson, Jennifer Piscitello, Ellyn Schmidt, Carolina Mallar, Bridget Davidson, Ruby Natale

**Affiliations:** 1 Center for Children and Families, Florida International University, Miami, USA; 2 Mailman Center for Child Development, Department of Pediatrics, University of Miami Miller School of Medicine, Miami, USA

**Keywords:** COVID-19, Coronavirus, Resilience, Risk, Caregiver well-being, Child mental health

## Abstract

**Background:**

Emerging work examining the psychological impact of COVID-19 on children and families suggests that the relationship between pandemic-related stress, child psychosocial functioning, and caregiver mental health are interrelated. However, much of this research is unidirectional and thus little is known about the bidirectional cascading effects children and caregivers may experience. The current study examined the transactional relationships between caregiver and child mental health over time during the COVID-19 pandemic.

**Methods:**

Linguistically, racially, and ethnically diverse caregivers (*N* = 286) of young children completed measures of caregiver mental health, caregiver pandemic-related stress, and child mental health (i.e., externalizing, internalizing, prosocial behavior) across three time points in the spring of 2020.

**Results:**

Using autoregressive cross-lagged analyses, impaired caregiver mental health at Time 1 (April 2020) predicted increased caregiver pandemic-related stress at Time 2 (May 2020). Caregiver pandemic-related stress at Time 1 predicted increased child internalizing symptoms at Time 2 which, in turn, predicted increased caregiver pandemic-related stress at Time 3 (July 2020). Lastly, impaired caregiver mental health at Time 2 (May 2020) predicted increased child externalizing symptoms at Time 3 (July 2020).

**Conclusions:**

Assessing transactional relationships between child and caregiver mental health during the COVID-19 pandemic is important to inform models of risk and resilience. Interventions at the level of the caregiver, the child, and/or the family should be considered as a way to interrupt potential negative developmental cascades.

## Introduction

The COVID-19 pandemic is a multisystem, cascading disaster that has produced negative impacts on young children and caregivers [1]. Although young children generally experience lower incidence of infection and mortality directly related to COVID-19 [2–4], recent work suggests that families with young children are particularly vulnerable to a host of negative outcomes given the level of interruption in essential services that promote child well-being and healthy development [5, 6]. These disruptions are ubiquitous, and deleterious effects have been documented for families across the globe. For example, interruptions in healthcare systems have resulted in delayed well-visits and reduced access to adequate medical care [7]. Increased rates of job loss and reductions in work hours have produced unprecedented financial strain on families [8, 9]. Protective resources typically available to families of young children have been closed or significantly altered, such as in-person education and closures of early learning and daycare facilities [10–12]. Beyond daily stressors, local and global governments and economies have been strained by the pandemic affecting the efficiency of various resources that individuals and families rely on (e.g., transportation, manufacturing, emergency, and other social services (e.g., child protective services), and humanitarian agencies) [1]. These disruptions are often associated with increased stress and negative mental health consequences among young children and their families immediately following the pandemic [13, 14], yet little is known about how children and caregivers are functioning over time.

Recent work suggests that pandemic-related stress and child psychosocial functioning are interrelated in their influence on caregiver’s mental health, such that child behavior problems and caregiver pandemic-related stress are associated with worse caregiver mental health outcomes [1, 5, 15, 16]. For example, higher rates of caregiver stress about the impacts of COVID on their housing, transportation, and finances as well as higher rates of child internalizing and externalizing problems were associated with high rates of caregiver mental health symptoms (e.g., anxiety, depression, sleep disturbance) [15]. Similarly, increased caregiver mental health symptoms (e.g., depression, anxiety) during the pandemic have been associated with increases in parent-reported child behavior problems [17]. However, most recently published studies examining pandemic-related functioning in families have largely been unidirectional or cross-sectional in design [15, 16, 18]. Research simultaneously testing the transactional relationship between child and caregiver stress and mental health over time is necessary to build empirically informed models of pandemic-related functioning and to inform intervention priorities and sequencing. Within the context of Family Systems Theory [19–22], as well as frameworks underlying family risk and resilience in disaster impacted samples [1, 23], the goal of this study was to examine the bidirectional relationship between pandemic-related stress and caregiver mental health and child behavior (i.e., internalizing, externalizing, and prosocial behaviors) over 4 months following the COVID-19 stay-at-home-order.

A larger body of work provides strong evidence for the reciprocal relationship between caregiver and child mental health [24–27]. For example, child externalizing symptoms, such as those associated with Attention-Deficit Hyperactivity Disorder and Oppositional Defiant Disorder, are associated with increased maternal depressive symptoms and in turn, maternal depression is associated with exacerbated externalizing problems over time [24, 28]. Thus, parents and children, alike, have the potential to exacerbate or reduce negative mental health consequences over the course of development. There is a paucity of work examining whether such transactional relationships exist during the current pandemic [29].

These bidirectional relationships can be understood within the context of Family Systems Theory and attachment theory. Family Systems Theory hypothesizes that the family unit is comprised of subsystems (e.g., co-caregiver unit, caregiver-child unit) and that disruptions in subsystems can impact the family as a whole [19–22]. Examining caregivers’ functioning within the broader family context provides insight about the complex relationships between family members [30]. This is also consistent with psychosocial models of recovery in other large-scale disasters that emphasize the importance of considering the social and societal circumstances impacting individual stress reactions [1, 23, 31]. Research examining naturally occurring cascades following natural disasters and related events may help inform models of pandemic functioning [32]. Further, attachment theory posits that parents’ ability to provide consistent and responsive caregiving (i.e., to facilitate secure attachment) is associated with improved parent–child relationships and a decreased risk for child psychopathology, while insecure attachment styles put children at risk for worse psychosocial outcomes [33]. Parental stress is associated with developing an insecure attachment [34] and given the context of the ongoing COVID-19 pandemic, may play an important role in the development of caregiver and child mental health and functioning [35]. Specifically, research during the COVID-19 pandemic found that parents with insecure attachment styles exhibited significantly higher negative emotions and perceived either fewer or greater negative emotions in their children, depending on the type of insecure attachment, supporting a body of work highlighting how caregiver emotion regulation influences children’s emotion regulation, particularly in the context of highly stressful situations [36].

In the context of natural disasters, caregiver psychopathology and poor family functioning are two of the most significant environmental risk factors impacting child adjustment [37–40]. Work in this area suggests that children of caregivers with the most severe responses have worse outcomes [37, 38, 41] but those caregivers who are well supported and engage in positive coping strategies may buffer the negative impact associated with disaster exposure on their children. For example, caregivers’ positive adjustment and use of parenting strategies such as warmth and acceptance have been found to be associated with decreases in depressive symptoms in children impacted by Hurricane Katrina [42]. Emerging research suggests similar findings in families negatively impacted by COVID-19 [15–17].

Results from unidirectional work is necessary to illuminate relevant variables associated with child and family pandemic-related functioning but may be misleading when the transactional nature of caregiver-child relationships is not considered. For example, it may be that children with increased internalizing and externalizing symptoms produce an additional co-occurring stressor for caregivers during a pandemic, impacting their mental health and stress-related functioning [13]. Alternatively, caregivers who experience high levels of stress and mental health symptoms may have a decreased ability to discuss emotions surrounding stressful events with their children [17], which can serve as a protective factor against mental health problems in children exposed to stressors [43]. A third possibility is that caregivers and children who demonstrate adaptive responses to the pandemic may influence each other to produce resilient outcomes [16]. Given the recency of COVID-19, a lack of longitudinal data precludes investigation of bidirectional relationships (or longitudinal relationships more broadly) in much of the early work on this topic. As a result, the directionality of these relationships has yet to be tested simultaneously within a longitudinal context. Therefore, the nuanced relationship between caregiver and child mental health functioning during the current pandemic is lacking.

Emerging work examining the impact of the COVID-19 pandemic and its associated disruptions on children and families has consistently documented the strong association between increased pandemic-related stressors and reductions in caregiver mental health functioning [5, 10, 15, 16, 44]. The increased levels of caregiver stress coupled with inadequate resources and support may negatively impact caregiver-child relationships and elevate risk for burnout or for more serious mental health consequences over time [5, 45]. Evidence suggests that negative caregiver reactions may have cascading impacts on child internalizing and externalizing symptoms [10, 18]. For example, increased pandemic stress has been associated with increased family conflict and use of negative parenting behaviors and in turn, increases in child distress and caregiver depression [16]. Conversely, positive coping (e.g., flexibility) is associated with potentially resilient pathways, such as increased family cohesion and use of positive parenting, resulting in positive child and caregiver outcomes [16]. Results of this early work suggests that caregivers can buffer the potential short-and long-term consequences associated with the current pandemic on their children [17]. Additional longitudinal work is necessary to disentangle the potential mechanisms that can be targeted to disrupt negative outcomes and promote positive adjustment in children and families [29, 46].

### Current study

The primary aim of this study was to evaluate the bidirectional relationship between pandemic-related stress, caregiver mental health functioning, and child behavior (i.e., internalizing, externalizing, and prosocial) across time in a racially, ethnically, and linguistically diverse sample of caregivers of young children. Our longitudinal study was designed to use cross-lagged analysis to evaluate the reciprocal relationship between caregiver and child mental health to inform future models of pandemic-related functioning in families of young children. We operationalized pandemic stress based on prior studies documenting effects of the pandemic on concerns regarding their health, the health of family members, employment, housing, transportation, having enough money for basic necessities, and relationships [47–49].

We tested the following hypotheses. First, we predicted a bidirectional relationship between caregiver mental health and pandemic-related stress with child externalizing symptoms such that impaired caregiver mental health and pandemic-related stress would predict worse child externalizing symptoms and child externalizing symptoms would, in turn, predict future caregiver mental health impairment and pandemic-related stress. Next, we predicted a bidirectional relationship between caregiver mental health and pandemic-related stress with child internalizing symptoms such that impaired caregiver mental health and pandemic-related stress would predict increases in future child internalizing symptoms, and this would, in turn, predict future caregiver mental health impairment and pandemic-related stress. Lastly, regarding resilience, we conducted an exploratory analysis to assess the bidirectional relationship between caregiver mental health, pandemic-related stress, and child prosocial behavior. We predicted a bidirectional relationship such that caregiver mental health impairment and pandemic-related stress would predict decreases in child prosocial behavior and vice versa. That is, increases in child prosocial behavior was also hypothesized to predict reduced future caregiver mental health impairment and pandemic-related stress.

## Method

### Participants

We recruited families from email lists of approximately 2000 caregivers participating in six service programs for children ages birth to 5 years from a university medical center in a metropolitan city in the Southeastern United States. This resulted in 260 participants from urban and suburban neighborhoods who completed the survey online. Using a community-based participatory research approach, 26 additional caregivers were recruited through a community partnership with a neighborhood center serving Haitian families (e.g., for food distribution). This resulted in a total sample of 286 caregivers of young children ages birth to 5 years, an adequate sample size to assess family functioning [50] while providing individualized follow-up support in response to needs expressed in each family survey. According to Kline (2015), determining minimum sample sizes for structural equation models (SEM) including for cross-lagged panel models is particularly difficult. However, median SEM sample sizes are around *N* = 200, and typically considered acceptable while sample sizes *N* < 100 are considered unacceptable [46]. See Table 1 for sample demographic information. The racial and ethnic makeup of the sample was representative of the broader county community, with approximately 85% of the sample being ethnic minorities. Twenty-four percent of families completed the survey in Spanish, and 3% completed the survey in Haitian Creole.

**Table 1:** Participant demographic characteristics

Families (N = 286)	% or M	n or SD
Caregiver age (range 18–54 years)	34.31	6.68
Caregiver gender		
Female	79.4%	227
Unknown/missing	12.9%	37
Male	7.7%	22
Caregiver ethnicity—Hispanic/Latinx	50.0%	143
Race		
White	17.50%	50
Black	15.7%	45
African American	14.30%	4
Asian/Pacific Islander	2.1%	6
Other	1.4%	4
Prefer not to respond	1.4%	4
Indian	1%	3
Native American/Indigenous	0.3%	1
Average number of children (range 1–7 children)	1.97	1.08
Child age	6.21	4.93
Survey language		
English	73.7%	191
Spanish	23.6%	61
Creole	2.7%	7

Child age calculated across all children, including multiple children within families

### Measures

In order to assess the functioning of families with young children during the COVID-19 pandemic, we developed a Risk and Resilience Survey based partially on previously validated measures. The survey included a section with family demographic information (see Table 1). Each measure was administered at all three time points.

#### Caregiver measures

*COVID-related Stress* The Everyday Stressors Index (ESI) [51] was used to evaluate caregivers’ level of concern regarding their health, the health of family members, employment, housing, transportation, having enough money for basic necessities, and relationships. The instructions were adapted to state, “The following are questions of common problems that people have related to their experience with the coronavirus/COVID-19 pandemic.” Respondents indicated their level of concern along a Likert scale ranging from (1) *not at all bothered*, (2) *a little bothered*, (3) *somewhat bothered*, (4) *bothered a great deal*, or (0) *don’t know*. The Everyday Stressors Index has demonstrated good reliability, validity, and internal consistency, including in samples of low-income families with young children [51, 52]. Cronbach’s alpha across time points ranged from 0.87 to 0.89. Two novel items were administered to assess concerns related to childcare and virtual schooling from home.

*Caregiver mental health symptoms* Caregiver mental health was assessed using selected items from the Experiences Related to COVID-19 Questionnaire [53], a scale piloted in the United States based on studies of adult and teenage stress responses following major traumatic events. This measure has been used by other researchers actively collecting and in the process of publishing studies focused on mental health and well-being, and it has been administered in nine countries as part of a NICHD-funded administrative supplement intended to advance understanding of the COVID pandemic [53, 54]. Furthermore, the use of a broad self-report measure of mental health symptoms is consistent with the approach taken by other researchers to examine the psychological impact of the COVID pandemic [55, 56] as well as other crises [57]. Participants used a four-point Likert scale ranging from “*strongly disagree*” to “*strongly agree*” to indicate worsened anxiety, anger, sadness/depression, eating, sleep, hopefulness about the future, and arguments since the start of the pandemic Cronbach’s alpha across time points ranged from 0.75 to 0.79. An additional item asked how personally disruptive the pandemic has been to daily routines, work, and family life from 1 (*not at all*) to 10 (*extremely*).

#### Child psychosocial concerns

The Strengths and Difficulties Questionnaire (SDQ) was used to screen for positive and negative psychological attributes in the child (limited to those older than age 2) who the caregiver perceived as having the most difficulty during the COVID-19 pandemic. Caregivers used a three-point Likert ranging from “not true” to “certainly true” to indicate attributes of their child’s personality and behavior. The SDQ has been shown to have strong psychometric properties, satisfactory reliability, and to be a useful measure of adjustment and psychopathology of pre-school and school-aged children [58, 59]. The internalizing (e.g., “Has many worries or often seems worried”, “Often unhappy, depressed, or tearful”), externalizing (e.g., “Often loses temper”, “Often argumentative with adults”), and prosocial (e.g., “Considerate of others’ feelings”, “Helpful if someone is hurt, upset, or feeling ill”) subscales were used in this study. Cronbach’s alpha for the internalizing subscale (0.68–0.69), the externalizing subscale (0.84–0.86) and the prosocial subscale (0.80–0.82.) were acceptable.

### Procedures

All procedures performed were approved by the university Institutional Review Board. The survey was emailed to families using REDCap, and was available in English, Spanish, and Creole. Due to concerns regarding email accessibility and literacy, a community partner site administered surveys in person. Informed consent was obtained either online or in person, depending on administration. At the first time point, the survey was open from April 22nd to May 22nd of 2020, during a Stay-at-Home order for the community. Responses were not anonymous for the purpose of providing follow-up support; however, participants could skip questions. Participants received electronic (emailed) or physical (in person) gift cards. At the second time point, the survey was emailed to all participants from the first time point on May 29th and was open until June 19th. At the third time point, the survey was emailed to all participants from the first time point on June 26th and was open until July 17th.

At all time points, survey responses from the REDCap database were compiled twice weekly by the study coordinator and sent to research staff who triaged follow-up support according to the urgency indicated by each caregiver. Resources and referrals provided were tailored according to the 7-tiered system of supports. Follow-up contacts included phone calls, emails, and/or text messages, depending on caregiver preference indicated in the survey.

### Analytic plan

To test hypotheses one through three, a series of cross-lagged path models [60] were constructed within *Mplus* 8 to examine the longitudinal associations between caregiver mental health impairment, pandemic-related stress, and child functioning (i.e., externalizing symptoms, internalizing symptoms, prosocial behaviors) at Time 1 (April 2020), Time 2 (May 2020), and Time 3 (July 2020). Autoregressive cross-lagged path models assess the relationship between variables across time such that change in variables across occasions are accounted for by regressing each repeatedly assessed variable on its immediate prior value. Additionally, the models simultaneously use cross-lagged, across-time, paths such that variable X at Time 1 predicts variable Y at Time 2, while controlling for variable Y at Time 1. Further, within time correlated errors between the variables were also modeled. Only forward paths were included in the models. Model fit was estimated using root mean square error of approximation (RMSEA; less than 0.05 is considered excellent fit), the comparative fit index (CFI), and the Tucker-Lewis index (TLI; values greater than 0.9 suggest excellent fit). To assess our three hypotheses, three cross-lagged path models were estimated: (1) caregiver mental health, caregiver pandemic-related stress, and child externalizing symptoms; (2) caregiver mental health, caregiver pandemic-related stress, and child internalizing symptoms, and (3) caregiver mental health, caregiver pandemic-related stress, and child prosocial behavior. Data were missing at random as indicated by nonsignificant Little MCAR test (Chi-square = 307.02, df = 319, *p* = 0.675). While there are limited recommendations as to what proportion of data is required to calculate composite scores in a given measure, conservative estimates were used which required that 80% of data in a given measure be available in order to calculate composite scores. However, one questionnaire had eight questions and thus, we rounded down to require that six out of eight questions were completed (i.e., 75% completed) in order to calculate the composite score. Missing data were imputed using maximum likelihood estimation with robust standard errors within *Mplus 8*. All models accounted for child age and parent ethnicity.

## Results

### Preliminary analyses

Descriptive statistics are reported in Table 2 and zero-order correlations among the study variables are reported in Table 3. Impaired caregiver mental health and caregiver pandemic-related stress were positively correlated across all three time points. Both impaired caregiver mental health and caregiver pandemic-related stress were positively correlated with child internalizing and externalizing symptoms across time such that worse caregiver mental health or stress was associated with worse child functioning across time. In contrast, impaired caregiver mental health and pandemic-related stress was largely unrelated to child prosocial behavior. Child internalizing and externalizing symptoms were positively correlated across time. Lastly, both child internalizing and externalizing were negatively correlated with child prosocial behavior such that higher levels of both internalizing and externalizing symptoms were related to less prosocial behavior across time.

**Table 2:** Descriptive statistics of variables of interest

Measure	n (N = 286)	Range of possible scores	Minimum score in sample	Maximum score in sample	M	SD
Everyday Stressors Index T1	286	0–80	7	72	37.52	11.77
Everyday Stressors Index T2	139	0–80	20	74	35.99	12.08
Everyday Stressors Index T3	164	0–80	0	69	35.90	11.77
Caregiver mental health symptoms T1	283	0–32	8	32	18.64	5.14
Caregiver mental health symptoms T2	139	0–32	8	31	18.39	5.48
Caregiver mental health symptoms T3	160	0–32	8	30	18.66	4.91
Child internalizing symptoms T1	182	0–20	0	15	4.10	3.33
Child internalizing symptoms T2	107	0–20	0	13	4.16	3.19
Child internalizing symptoms T3	125	0–20	0	20	6.38	4.46
Child externalizing symptoms T1	181	0–20	0	20	8.5	5.03
Child externalizing symptoms T2	107	0–20	0	18	8.14	4.67
Child externalizing symptoms T3	125	0–20	0	20	8.50	4.48
Child prosocial behavior T1	182	0–10	0	10	6.47	2.87
Child prosocial behavior T2	107	0–10	0	10	6.57	2.63
Child prosocial behavior T3	125	0–10	0	10	6.66	2.73

**Table 3:** Zero-order correlations between caregiver mental health, pandemic-related stress, child externalizing, child internalizing, and prosocial behaviors

	1	2	3	4	5	6	7	8	9	10	11	12	13	14	15
1. CG MH T1	–	.71**	.51**	.57**	.54**	.39**	.34**	.27*	.24*	.41**	.34**	**.22**	-.28**	.17	.02
2. CG MH T2		–	.54**	.49**	.66**	.49**	.34**	.38**	.44**	.34**	.36**	.42**	-.16	-.12	-.03
3. CG MH T3			–	.27**	.43**	.43**	**.24**	.20	.27**	**.25**	.32**	.24*	−.14	−.15	.01
4. CG PS T1				–	.63**	.45**	.36**	.27*	.32**	.40**	.37**	.29**	−.11	−.10	.00
5. CG PS T2					–	.65**	.36**	.33**	.33**	.32**	.43**	.30*	−.08	−.07	.01
6. CG PS T3						–	**.25**	.14	.30**	**.25**	.35**	**.21**	.01	.03	.00
7. Child Ext T1							–	.70**	.70**	.59**	.52**	.58**	-.40**	-.32*	-.31*
8. Child Ext T2								–	.80**	.40**	.56**	.61**	-.41**	-.33**	**-.27**
9. Child Ext T3									–	.50**	.64**	.84**	-.40**	-.34*	-.32**
10. Child Int T1										–	.61**	.51**	-.44**	-.32*	-.38**
11. Child Int T2											–	.58**	-.45**	-.36**	**-.26**
12. Child Int T3												–	-.46**	-.38**	-.33**
13. Child Pro T1													–	.74**	.69**
14. Child Pro T2														–	.72**
15. Child Pro T3															–

CG: caregiver; MH: mental health; PS: pandemic-related stress; Ext: externalizing symptoms; Int: internalizing symptoms; Pro: prosocial behaviors; T1: time 1; T2: time 2, T3: time 3

### Is there a bidirectional relationship between impaired caregiver mental health, caregiver pandemic-related stress, and child externalizing symptoms?

**Fig. 1:**
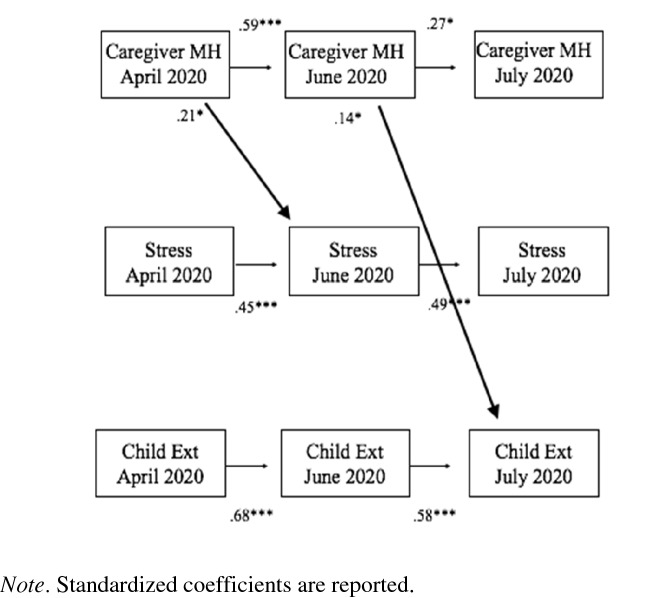
Relationship between caregiver pandemic-related stress, caregiver mental health, and child externalizing symptoms. Standardized coefficients are reported. All cross-lagged paths and within-time correlated errors are modeled, but not displayed for clarity. See Table 4 for all parameter estimates

The results of the first cross-lagged model testing the associations among impaired caregiver mental health, caregiver pandemic-related stress, and child externalizing symptoms are presented in Table 4, Fig. 1. The model fit the data well (Chi-Square (6) = 5.31, *p* = 0.505; RMSEA = 0.00; CFI./TLI = 1.0/1.01; SRMR = 0.01). The stability paths were significant for all variables (*B*s = 0.27-0.68, *p*s < 0.001), suggesting moderate to high levels of stability of caregiver mental health, pandemic-related stress, and child externalizing symptoms over time. Caregiver mental health at Time 1 predicted caregiver pandemic-related stress at Time 2 such that impaired caregiver mental health predicted worse future pandemic-related stress (*B* = 0.21, *p* < 0.05)*.* In addition, impaired caregiver mental health at Time 2 predicted worse child externalizing symptoms at Time 3 (*B* = 0.14, *p* < 0.05).

**Table 4:** Standardized estimates for cross-lagged path model assessing the bidirectional relationship between caregiver pandemic-related stress, caregiver mental health, and child externalizing symptoms

Measure	Estimate	SE	p
Autoregressive coefficients			
Caregiver stress T1 → Caregiver stress T2	.45***	.11	.001
Caregiver stress T2 → Caregiver stress T3	.49***	.14	.001
Caregiver MH T1 → Caregiver MH T2	.59***	.09	.001
Caregiver MH T2 → Caregiver MH T3	.27*	.12	.023
Child externalizing T1 → Child Externalizing T2	.68***	.07	.001
Child Externalizing T2 → Child Externalizing T3	.58***	.09	.001
Predicting caregiver MH			
Caregiver stress T1 → Caregiver MH T2	.09	.09	.286
Caregiver stress T2 → Caregiver MH T3	.10	.12	.417
Child Externalizing T1 → Caregiver MH T2	.10	.08	.216
Child Externalizing T2 → Caregiver MH T3	-.07	.07	.307
Predicting caregiver stress			
Caregiver MH T1 → Caregiver stress T2	.21*	.10	.039
Caregiver MH T2 → Caregiver stress T3	.08	.09	.402
Child externalizing T1 → Caregiver stress T2	.10	.08	.209
Child externalizing T2 → Caregiver stress T3	-.02	.11	.879
Predicting child externalizing			
Caregiver MH T1 → Child externalizing T2	-.05	.08	.521
Caregiver MH T2 → Child externalizing T3	.14*	.07	.041
Caregiver stress T1 → Child externalizing T2	.08	.10	.452
Caregiver stress T2 → Child externalizing T3	-.11	.09	.192

Note. T1 = Time 1. T2 = Time 2. T3 = Time 3. MH = mental health. Models controlled for ethnicity and age of child

### Is there a bidirectional relationship between caregiver mental health, caregiver pandemic-related stress, and child internalizing symptoms?

**Fig. 2:**
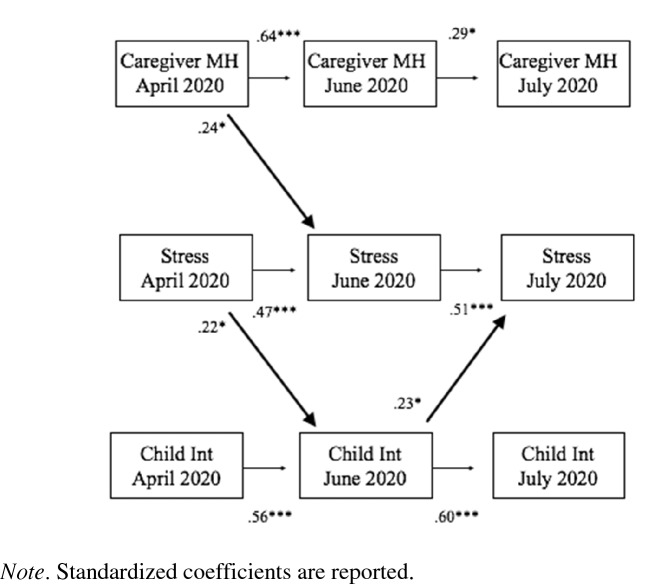
Relationship between caregiver pandemic-related stress, caregiver mental health, and child internalizing symptoms. Standardized coefficients are reported. All cross-lagged paths and within-time correlated errors are modeled, but not displayed for clarity. See Table 5 for all parameter estimates

The results of the second cross-lagged model tested the associations among impaired caregiver mental health, caregiver pandemic-related stress, and child internalizing symptoms are presented in Table 5 and Fig. 2. The model fit the data well (Chi-Square (14) = 20.27, *p* = 0.12; RMSEA = 0.04; CFI./TLI = 0.99/0.95; SRMR = 0.04). The stability paths were significant for child internalizing symptoms (*B*s = 0.56 − 0.60, *p*s < 0.001), suggesting moderate to high levels of stability of child internalizing symptoms over time. As shown in Table 5 and Fig. 2, and consistent with hypotheses, there was a bidirectional relationship between caregiver pandemic-related stress and child internalizing symptoms. Specifically, caregiver pandemic-related stress at Time 1 predicted increases in child internalizing symptoms at Time 2 (*B* = 0.22, *p* < 0.05), which then, in turn, predicted increases in caregiver pandemic-related stress at Time 3 (*B* = 0.23, *p* < 0.05). In addition, as with the first cross-lagged model, impaired caregiver mental health at Time 1 predicted worse caregiver pandemic-related stress at Time 2 *(B* = 0.24, *p* < 0.05).

**Table 5:** Standardized estimates for cross-lagged path model assessing the bidirectional relationship between caregiver pandemic-related stress, caregiver mental health, and child internalizing symptoms

Measure	Estimate	SE	*p*
Autoregressive coefficients			
Caregiver stress T1 → Caregiver stress T2	.47***	.08	.001
Caregiver stress T2 → Caregiver stress T3	.51***	.09	.001
Caregiver MH T1 → Caregiver MH T2	.64***	.07	.001
Caregiver MH T2 → Caregiver MH T3	.29***	.12	.016
Child Internalizing T1 → Child internalizing T2	.56***	.07	.001
Child internalizing T2 → Child internalizing T3	.60***	.08	.001
Predicting caregiver MH			
Caregiver stress T1 → Caregiver MH T2	.11	.08	.165
Caregiver stress T2 → Caregiver MH T3	.12	.10	.239
Child internalizing T1 → Caregiver MH T2	-.02	.08	.842
Child internalizing T2 → Caregiver MH T3	.02	.09	.822
Predicting Caregiver stress			
Caregiver MH T1 → Caregiver stress T2	.24**	.09	.006
Caregiver MH T2 → Caregiver stress T3	.07	.10	.491
Child internalizing T1 → Caregiver stress T2	.01	.09	.900
Child internalizing T2 → Caregiver stress T3	.23*	.10	.019
Predicting child internalizing			
Caregiver MH T1 → Child internalizing T2	-.04	.09	.612
Caregiver MH T2 → Child internalizing T3	.15	.10	.133
Caregiver stress T1 → Child internalizing T2	.22*	.09	.011
Caregiver stress T2 → Child internalizing T3	-.02	.11	.887

Note. T1 = Time 1. T2 = Time 2. T3 = Time 3. MH = mental health. Models controlled for ethnicity and age of child

### Is there a bidirectional relationship between impaired caregiver mental health, caregiver pandemic-related stress, and child prosocial behavior?

**Fig. 3:**
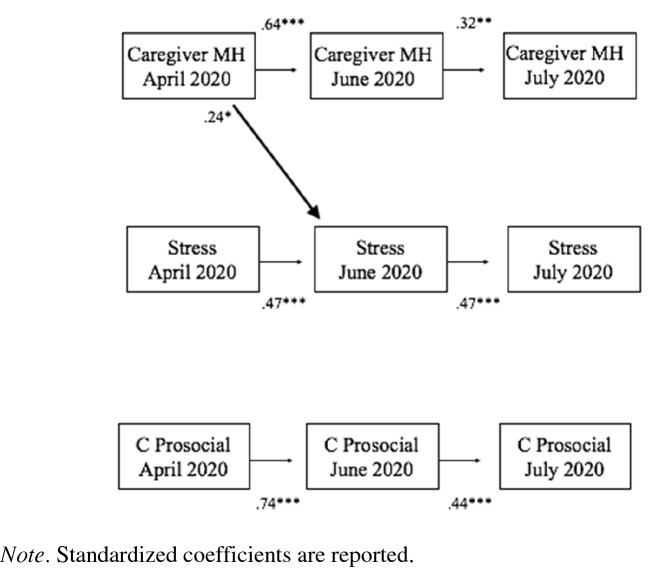
Relationship between caregiver pandemic-related stress, caregiver mental health, and child prosocial behavior. Standardized coefficients are reported. All cross-lagged paths and within-time correlated errors are modeled, but not displayed for clarity. See Table 6 for all parameter estimates

The last cross-lagged model, which tested the associations among caregiver mental health, caregiver pandemic-related stress, and child prosocial behavior, is presented in Table 6 and Fig. 3. The model fit the data well (Chi-Square (10) = 6.97, *p* = 0.728; RMSEA = 0.00; CFI./TLI = 1.00/1.04; SRMR = 0.02). The stability paths were significant for child prosocial behavior (*B*s = 0.44–0.74, *p*s < 0.001), suggesting moderate to high stability of child prosocial behaviors over time. Contrary to hypotheses, there were no significant cross-lagged paths between these constructs with one exception. Like the previous models reported above, caregiver mental health at Time 1 predicted caregiver pandemic-related stress at Time 2 such that impaired caregiver mental health predicted worse future pandemic-related stress (*B* = 0.24, *p* < 0.05)*.*

**Table 6:** Standardized estimates for cross-lagged path model assessing the bidirectional relationship between caregiver pandemic-related stress, caregiver mental health, and child prosocial behavior

Measure	Estimate	SE	p
Autoregressive coefficients			
Caregiver stress T1 → Caregiver stress T2	.47***	.11	.001
Caregiver stress T2 → Caregiver stress T3	.47***	.13	.001
Caregiver MH T1 → Caregiver MH T2	.64***	.08	.001
Caregiver MH T2 → Caregiver MH T3	.32**	.11	.005
Child prosocial T1 → Child prosocial T2	.74***	.06	.001
Child prosocial T2 → Child prosocial T3	.44***	.13	.001
Predicting caregiver MH			
Caregiver stress T1 → Caregiver MH T2	.10	.09	.228
Caregiver stress T2 → Caregiver MH T3	.14	.12	.247
Child prosocial T1 → Caregiver MH T2	.00	.07	.996
Child prosocial T2 → Caregiver MH T3	-.05	.07	.428
Predicting caregiver stress			
Caregiver MH T1 → Caregiver stress T2	.24*	.11	.026
Caregiver MH T2 → Caregiver stress T3	.08	.09	.382
Child prosocial T1 → Caregiver stress T2	-.01	.08	.922
Child prosocial T2 → Caregiver stress T3	-.04	.08	.648
Predicting child prosocial behaviors			
Caregiver MH T1 → Child prosocial T2	.06	.09	.480
Caregiver MH T2 → Child prosocial T3	.14	.09	.135
Caregiver stress T1 → Child prosocial T2	-.09	.09	.313
Caregiver stress T2 → Child prosocial T3	-.04	.11	.699

Note. T1 = Time 1. T2 = Time 2. T3 = Time 3. MH = mental health Models controlled for ethnicity and age of child

## Discussion

The primary aim of this study was to evaluate the transactional relationship between caregiver and child functioning from April–July 2020 during the COVID-19 pandemic among diverse families living in a U.S. metropolitan. In line with Family Systems Theory [19–22], as well as frameworks underlying family risk and resilience [1, 23], we hypothesized that impaired caregiver mental health and pandemic-related stress would predict worse child externalizing symptoms and these symptoms would, in turn, predict worse future caregiver mental health impairment and pandemic-related stress. Similarly, we also hypothesized that caregiver mental health impairment and pandemic-related stress would predict worse child internalizing symptoms and these symptoms would, in turn, predict worse future caregiver mental health impairment and pandemic-related stress. Regarding resilience, we predicted that reduced caregiver mental health impairment and pandemic-related stress would predict increased child prosocial behavior, and this would, in turn, predict reduced future caregiver mental health impairment and pandemic-related stress. The current study adds to and extends the literature on the transactional relationships between caregiver and child functioning during the global COVID-19 pandemic.

Our first hypothesis predicting a bidirectional relationship between caregiver mental health, pandemic-related stress, and child externalizing symptoms was partially supported. Specifically, worsened caregiver anxiety, anger, sadness/depression, eating, sleeping, hopefulness about the future, and arguments since the beginning of COVID-19 pandemic predicted the severity of their children’s temper, arguments, and hyperactivity one month later. These findings are generally consistent with work demonstrating that worse parent mental health increase childhood behavior problems [61, 62]. Although parent–child attachment was not assessed directly, it is possible that high levels of caregiver stress during the pandemic negatively impacted parent–child attachment, which may have resulted in increased risk for behavior problems [63].

However, child behavior problems did not predict increased caregiver mental health concerns, nor did we find any bidirectional effects of COVID-19 pandemic-related stress on child behavior problems. These findings are somewhat surprising given work demonstrating the transactional nature of caregiver functioning and child externalizing symptoms within the larger body of literature [24, 27]. Notably, our sample’s baseline externalizing symptoms were in the mild to moderate range, suggesting that our sample may not have clinically significant levels of externalizing symptoms, where the severity of the symptoms may prove more stressful for caregivers. Future work may choose to examine these relationships in a clinical sample or investigate potential individual differences (e.g., children who are high or low on measures of externalizing symptoms). In addition, it is important to highlight that the stability paths suggest both caregiver and child functioning remained stable over time, a promising finding that suggests functioning, on average, did not deteriorate further as the pandemic continued.

Our second hypothesis predicting a bidirectional relationship between caregiver mental health, pandemic-related stress, and child internalizing symptoms was also partially supported. Caregiver mental health impairment did not predict child internalizing symptoms or vice versa. However, caregiver stress regarding their health, the health of family members, employment, housing, transportation, having enough money for basic necessities, and relationships led to increases in their children being worried, tearful, or sad one month later. Critically, their children’s worry and sadness lead to compounded future caregiver pandemic-related stress regarding health, employment, housing, finances, and relationships. These findings are in line with the broader literature [62, 64] and with other findings during the COVID-19 pandemic [17] demonstrating the impact of caregivers’ stress on children’s internalizing symptoms. As was found with children’s externalizing symptoms, children’s internalizing symptoms were stable across time suggesting functioning, on average, did not deteriorate further as the pandemic progressed [27] and extends the literature by demonstrating the transactional nature of these relationships during the COVID-19 pandemic. Specifically, children may become more aware of parental stress through methods such as overhearing adult conversations that may influence their level of worry. Another possibility is that parents who are under significant stress may be less available to help their children manage their own stress responses or strengthen their coping skills [17], consistent with attachment theory [33].

Lastly, our third hypothesis that predicted a bidirectional relationship between caregiver mental health, pandemic-related stress, and child prosocial behavior was not supported, as we found no relationship between these constructs. Specifically, improved mental health among caregivers did not predict improvements in their children’s ability to be considerate of others’ feelings or helpful if someone is hurt, upset, or feeling ill, or vice versa. Perhaps this finding is unsurprising given that caregiver mental health or stress was unrelated to child prosocial behavior across time when examined using Pearson’s correlation. In line with models of post-disaster resilience in families [1, 23, 37], future work may consider focusing on how resilience in parents (not just the absence of pathology) is related to resilience in children. Research supports the cascading relationships between these variables and it may be that a similar pattern will emerge during the current pandemic. Again, as with previous models, prosocial behavior showed moderate to high stability over time suggesting these behaviors, on average, did not deteriorate as the pandemic progressed.

Consistently across all three models, caregiver mental health impairment (e.g., worse caregiver anxiety, anger) predicted increases in future caregiver pandemic-related stress. Interestingly, caregiver pandemic-related stress did not predict increases in future caregiver mental health concerns. This finding suggests that alleviating caregivers’ mental health concerns early on would reduce pandemic-related stress in the future, but not vice versa, informing the sequencing of interventions for caregivers of young children during the COVID-19 global pandemic.

### Clinical implications

Given the extensive disruptions to all areas of life that COVID-19 introduced for families, appropriately assessing and triaging families who are most in need of support is important. The ability to distinguish families who are experiencing natural reactions to stress versus more severe symptoms of anxiety, depression, post-traumatic stress, and adjustment disorders is critical for using resources most appropriately and providing services to those most in need. The current study has important treatment implications for caregivers and their children impacted by the COVID-19 pandemic. First, our data suggest that interventions for caregiver’s mental health immediately following the start of a disruptive occurrence such as a pandemic would reduce the level of stress felt by caregivers. For example, psychological interventions that teach behavioral and cognitive coping strategies and are effective at reducing symptoms of mental health problems including anxiety and depression may be especially important at the onset of a disaster. Second, our data suggest that providing resources to reduce caregiver mental health concerns and stress is likely to reduce the risk of behavior problems and distress in their children, respectively, and that targeting children’s worry and distress with interventions may reduce the compounding, co-occurring stress felt by caregivers. For example, psychological interventions such as mindfulness meditation and cognitive behavior therapy are effective at reducing stress and anxiety in adults and children, respectively [65, 66].

### Strengths, limitations, and future directions

The current study had several strengths. First, we collected data on child and caregiver functioning in the immediate aftermath of COVID-19 lockdowns in the United States, providing a longitudinal picture of pandemic functioning in families. Second, the study included a racially, ethnically, and linguistically diverse sample of families in a metropolitan area, making our findings of the relationships between caregiver and child functioning generalizable to a broad range of families. Critically, an additional important methodological advance of the current study is the longitudinal design using three months of data following the national lockdown. This methodology is important as very little research on the pandemic has utilized longitudinal data [29], and because it allowed us to use autoregressive cross-lagged models to test the bidirectional relationship between caregiver mental health, pandemic-related stress, and child functioning over multiple time periods, while simultaneously accounting for their correlational relationship within time points. This work contributes to the current literature to inform empirically supported models of long-term pandemic functioning.

Despite these strengths, the results of the current study need to be interpreted in light of some important limitations. First, while we captured caregiver and child functioning during the first several months of the earlier phase of the pandemic and lockdown in the United States, we did not capture levels of functioning before the March 2020 lockdown. Thus, we were unable to assess the immediate, acute impacts of the pandemic. Additionally, our final time point was in July 2020, and the impacts of the pandemic on child and caregiver functioning have likely been amplified in the subsequent months, as COVID-19 has continued to disrupt the lives of families in the year since these data were collected. Longer-term impacts of the pandemic on child and caregiver functioning may differ from the immediate impacts that we measured, particularly given the substantial sense of loss, either of the caregiver themselves or the psychosocial losses with prolonged lockdowns, restrictions, and limited social engagements. We were also unable to include data on predisposing factors such as caregiver and child mental health prior to the start of the pandemic and we did not directly assess parent–child attachment, which likely plays an important role in the outcomes of families with young children during this stressful time. For example, it may be that secure attachment style prior to the pandemic served as a protective factor and buffered the potential negative consequences of worsened caregiver mental health functioning on child functioning, and vice versa. Further, we were limited in our ability to use a standardized measure of resilience or measures of other risk factors (i.e., family dysfunction) that may influence the relationships between our constructs.

In addition, given that families who were most impacted by the pandemic may not have participated in our study or may have dropped out due to pandemic-related stress, it is possible our findings may not have captured the most vulnerable of families. Regarding the choice of measures, using a broad band measure of caregiver mental health may have limited our ability to identify relationships between child and caregiver functioning and understand which aspects of caregiver mental health were most critical to child functioning. That is, the relationships between child and caregiver functioning may possibly differ with the use of narrow band measures (e.g., depression, anxiety). Moreover, our measure of prosocial behavior included an individual item ‘shares readily with others.’ Given social distancing recommendations, this item may have artificially deflated the prosocial behavior subscale such that children may have been identified as having had more difficulties than they would under circumstances in which social interaction was more permissible. Indeed, approximately 20% of caregivers rated that their child never shares readily with others.

Generalizability of these results should be interpreted with caution given that the sample—albeit ethnically and linguistically diverse—is limited in that these data represent the experience of families in a large southeastern city in the United States. A localized lockdown across the United States indicate that our results likely apply to families in other regions of the United States since most schools, places of employment, and daily activities were interrupted within a similar time frame. Although local and national lockdowns following the identification of positive COVID-19 cases varied widely across countries [67], the results of the present study may generalize to families across the globe who were disrupted by COVID-19 lockdowns in similar ways. However, significant cultural and societal differences between countries must be acknowledged and therefore may limit the generalizability of findings to families living in countries outside the United States. For example, financial burdens experienced by families may have been offset by governmental support, such as income supplement or debt relief, which may have ranged significantly between countries and may have significantly influenced the stress felt by caregivers.

### Future directions

Given research suggesting that family dysfunction is one of the most significant environmental risk factors impacting child adjustment following a natural disaster [37], research should also incorporate this in future models as family dysfunction may mediate the relationship between caregiver and child functioning [16]. As discussed above, future research should assess how caregiver attachment mediates or moderates the relationships between caregiver functioning and child functioning within the context of the current disaster. Given the potential for cultural differences impacting the results of the current study, future work should attempt to replicate the current study in countries and cultures outside of the United States. Finally, given our results demonstrating that parental pandemic-related stress increases future child internalizing symptoms and these symptoms, in turn, predict increased future caregiver stress, future research should examine whether interventions that target caregiver stress and children’s internalizing symptoms directly result in decreases in these symptoms.

### Conclusions

Given the ongoing nature of the COVID-19 pandemic, it is likely that many families have and will continue to experience stress and negative health and mental health consequences. While some will experience common stress reactions that resolve spontaneously, there may be a subset of families who will experience clinically significant symptoms as a result of the pandemic and may benefit from targeted interventions. In our sample, greater caregiver pandemic-related stress predicted worse child internalizing symptoms and these internalizing symptoms, in turn, predicted additional future caregiver pandemic-related stress, highlighting the transactional dynamics of psychosocial wellbeing between caregivers and their children. Thus, interventions at the level of the caregiver, the child, and/or the family should be considered as a way to interrupt potential negative developmental cascades [68].

## Data Availability

The datasets used and/or analyzed during the current study are available from the corresponding author on request.

## Abbreviations

RMSEA: Root mean square error of approximation
CFI: Comparative fit index
TLI: Tucker-Lewis index
MCAR: Missing completely at random
